# Biological Relevance of Dual Lysine and N-Terminal Methyltransferase METTL13

**DOI:** 10.3390/biom14091112

**Published:** 2024-09-03

**Authors:** Mullen Boulter, Kyle K. Biggar

**Affiliations:** Institute of Biochemistry, Carleton University, 1125 Colonel By Drive, Ottawa, ON K1N 5B6, Canada; mullenboulter@cmail.carleton.ca

**Keywords:** post-translational modification (PTM), methyltransferase (MTase), lysine methylation, N-terminal methylation, METTL13

## Abstract

The dual methyltransferase methyltransferase-like protein 13, also referred to as METTL13, or formerly known as FEAT (faintly expressed in healthy tissues, aberrantly overexpressed in tumors), has garnered attention as a significant enzyme in various cancer types, as evidenced by prior literature reviews. Recent studies have shed light on new potential roles for METTL13, hinting at its promise as a therapeutic target. This review aims to delve into the multifaceted biology of METTL13, elucidating its proposed mechanisms of action, regulatory pathways, and its implications in disease states, as supported by the current body of literature. Furthermore, the review will highlight emerging trends and gaps in our understanding of METTL13, paving the way for future research efforts. By contextualizing METTL13 within the broader landscape of cancer biology and therapeutics, this study serves as an introductory guide to METTL13, aiming to provide readers with a thorough understanding of its role in disease phenotypes.

## 1. Introduction

Post-translational modifications (PTMs) are covalent modifications made via protein–protein interactions that control the activation and deactivation of cellular pathways. To date, roughly 400 distinct PTMs have been identified [1]. PMTs have been observed on both histone and non-histone targets and are involved in a wide variety of cellular processes, including signal transduction, the control of processes such as protein synthesis and chromatin organization, and disease phenotype presentation [2,3]. There are three generalized categories of enzymes which catalyze PTMs, including “writers”, which are enzymes that carry out the chemical modification of a substrate, “readers”, which are enzymes that contain a recognition domain involved in identifying the modification carried out by the “writer” enzymes, leading to the downstream changes in signaling based on the PTM, and “erasers”, which are the enzymes responsible for reversing or removing the PTM [3,4]. An enzyme can have multiple functions as well, serving simultaneously as any of the roles mentioned above for different targets. Commonly studied PTMs include acetylation, ubiquitination, phosphorylation, and methylation [2,3]. Our understanding of PTMs and their impact on protein biology has extended the known functional human proteome. PTMs act as modulators of protein activity, making them a very valuable target for therapeutic development.

The rate and occurrence of PTMs change in disease phenotypes, suggesting that specific PTMs may be used as biomarkers for disease prognosis [3]. Cells which form cancerous tumors display different growth pathways, are more resistant to apoptosis, and display higher levels of mechanical stress in comparison to their non-cancerous counterparts. This is in part due to differences in PTM-mediated regulatory pathways [3]. As such, specific PTMs have been characterized as being involved in tumorigenesis, apoptosis resistance, and malignancy; however, only a small fraction of identified PTMs have been studied extensively in the context of disease progression [3]. This remains an active and important area of research to better understand how disease phenotypes manifest. Additionally, targeting disease phenotype PTMs has been suggested as a promising avenue for therapeutic development [5].

One notable PTM is protein methylation, which entails the transfer of a methyl group (-CH3) from a methyl donor, S-adenosylmethionine (SAM), to a target. Enzymes that catalyze methyl transfer are referred to as methyltransferases (MTases). Methyl transfer can occur on the side chains of amino acid residues such as lysine, arginine [6,7,8], histidine [9], glutamate, and aspartate [10]. Additionally, the N-terminal of proteins can undergo methylation either directly on the amino group of methionine or on the amino group of the following amino acid, assuming methionine is cleaved via methionine aminopeptidases [11,12]. Focusing primarily on lysine and N-terminal methylation, these enzymes are designated as lysine methyltransferases (KMTs) and N-terminal methyltransferases (NTMTs), respectively [8,11,12]. Lysine methyl transfer is known to be a reversible process, with demethylation catalyzed by enzymes referred to as lysine demethylases (KDMs); however, to date N-terminal methylation is thought to be an irreversible process, and no validated N-terminal demethylases have been identified [11]. 

There are two main structural classes of KMTs, including the suppressor of variegation 3–9, the enhancer of zeste, and Trithorax (SET)-domain-containing KMTs and seven-beta-strand (7βS) KMTs. SET KMTs are typically responsible for histone lysine methylation events, and 7βS KMTs are currently known to be responsible for non-histone lysine targets [13]; however, exceptions do exist for both classes as more validated substrates are documented. The SET-domain-containing class contains roughly 50 known KMTs, while the 7βS KMT class includes over 130 enzymes, making it the largest class of MTases in the human proteome [13]. 

The mechanism of methyl transfer of the SET-domain-containing class has been characterized in much greater detail than that of the 7βS class [14,15]. Although, in general, methyl transfer is assumed to follow an SN2 reaction mechanism, the catalytic binding regions of the two classes differ greatly, suggesting that the two may use different binding mechanisms [14]. A computational study has modeled methyl transfer in 7βS KMT DOTL1 and suggested that the mechanism of methyl transfer differs greatly from that of the SET-domain-containing KMTs in terms of SAM-binding orientation, as well as substrate coordination [14,16]. There is very little structure homology within the 7βS class, so making generalized claims regarding the structural classes’ mechanisms based on the computational modelling of DOTL1 is not possible [14]. This, however, highlights the importance of additional research regarding structural interactions and determining binding mechanisms among 7βS KMTs.

The general process of methyl transfer is the same, whether catalyzed by a KMT or an NTMT. The MTase binds a substrate and SAM, the methyl donor, and catalyzes the transfer of a methyl group, resulting in the formation of S-adenosylhomocysteine (SAH), the demethylated form of SAM, and a methylated substrate [8,12]. Both the ε amino group of lysine and the α amino group of an N-terminal can occupy various methylation states. These are the addition of a single methyl-group (monomethylation; me1), the addition of two methyl-groups (dimethylation; me2), or the addition of three methyl-groups (trimethylation; me3). Increasing the number of methyl groups on a residue increases both the hydrophobic character of the methylated substrate and the steric bulkiness, influencing possible interactions. Trimethylation also renders the methylated amino group chemically inert by inducing a permanent positive charge and chemical saturation [8]. It is assumed that N-terminal methylation protects against protein degradation by inhibiting N-terminal acetylation or ubiquitination [11,12]. 

Methyltransferase-like protein 13 (METTL13) is an enzyme capable of both lysine and N-terminal methylation, rendering it a dual methyltransferase, and is the subject of the remainder of this review.

## 2. Structure and Function of METTL13

METTL13 is a methyltransferase with two distinct seven-beta-strand (7βS) catalytic domains responsible for catalyzing the SAM-dependent methylation of both isoforms of the eukaryotic elongation factor (eEFIA), eEFIA1 and eEFIA2, at two sites: lysine 55 and the N-terminal glycine [17]. Contextually, eEFIA1 is the more prevalent isoform, present in all cells, whereas eEFIA2 is typically only present in post-mitotic cells such as myocytes and neurons. Both isoforms, however, are active in cancerous cells [17,18]. Broadly, eEFIA is a GTPase responsible for delivering aminoacyl-transfer RNA molecules to the ‘A site’ of a ribosome for protein translation [8,17]. When METTL13 was initially identified as a novel enzyme involved in tumorigenesis, it was first given the name faint expression in normal tissues, aberrant overexpression in tumors (FEATs) [19]. This name highlights a very important point regarding METTL13 biology in disease phenotypes: treatments targeting METTL13 that result in decreased cell proliferation in cancerous cells typically do not result in decreased cell proliferation of healthy cells [19,20]. Although there is currently no targeted inhibitor for METTL13, inhibitor development for numerous MTases has begun [4,21], with some currently in stages of clinical trials [22,23,24]. These advancements, along with METTL13 expression levels in healthy versus tumor cells, highlight METTL13 as a promising therapeutic target. 

The subsequent study of the structure and function of METTL13 demonstrated that each catalytic domain is responsible for just one of the currently identified methylation events [17]. The N-terminal domain (MT13-N), which encompasses residues 46 through 160, catalyzes the dimethylation of lysine 55, and the C-terminal domain (MT13-C), encompassing residues 499 through 673, catalyzes the trimethylation of the N-terminal [25]. These domains are structurally unique from each other, and each can function both in vitro and in vivo as orphan enzymes [17]. It is important to note that the residues contained within each isolated domain may vary among researchers; however, the residues given above result in the retention of the enzymatic function, providing a general size of each domain. 

METTL13 is known to regulate the eEFIA function. Knockout (KO) experiments have demonstrated that removing METTL13 from cells affects the occupancy levels of different amino acid codons. METTL13 KO increases the occupancy of all lysine and histidine codons, as well as [ACG] for threonine, [CCG] for proline, and [AGG] for arginine, resulting in a slower rate of translation for these codons. Conversely, METTL13 KO decreases the occupancy of all alanine codons, as well as [TGG] and [TAC] for tryptophan, [AAC] for aspartic acid, and [TCA] and [TCC] for serine. This means that these codons are translated faster in the absence of METTL13 [17]. This clearly demonstrates that the absence of METTL13 has a direct effect on the rate of protein translation. 

**Figure 1:**
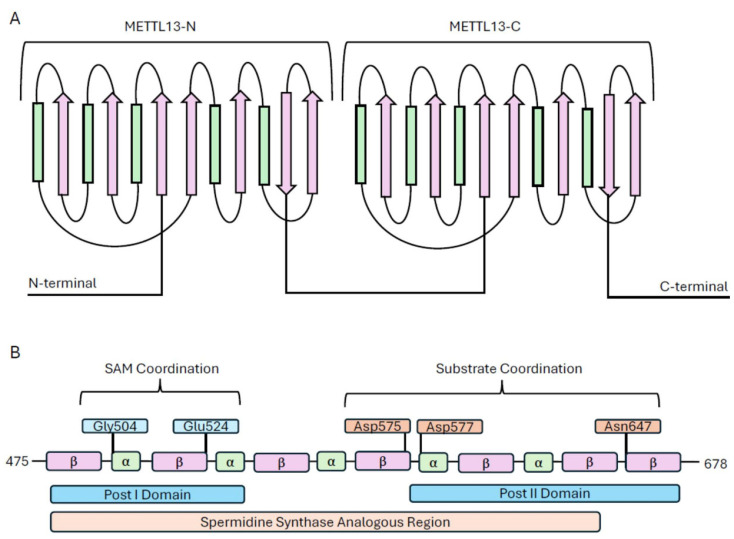
(**A**) Illustration of wild-type METTL13 with both catalytic domains, METTL13-N and METTL13-C, labelled. Rectangles, shown in green, represent alpha helices, and the arrows, shown in pink, represent beta sheets. Illustration adapted from Falnes et al. [13]. (**B**) Illustrated block diagram of METTL13-C with annotations for amino acids of interest in catalytic activity [8,17].

As stated, both METTL13 domains are 7βS methyltransferases, and METTL13, to date, is the only known member of the 7βS family, which contains two distinct catalytic domains. This classification is made based on the presence of distinct structural features, including the Rossmann fold characteristics, as well as similarities in SAM binding regions [8]. A schematic visualization of both METTL13 domains is shown in Figure 1A. Although many 7βS enzymes show these key structural features, there is very little structural homology in the family. Even the two subunits of METTL13 are not closely related: MT13-N most closely resembles other 7βS KMTs, including eEFIA1-KMT2, eEFIA1-KMT4, and METTL10, whereas MT13-C most closely resembles spermidine synthase, which is not a methyltransferase [8]. Enzymes belonging to this class have seven alternating beta strands in series, separated by alpha helices [8]. Other relevant structural features include the presence of the Post I and Post II domains, which are involved in SAM coordination and substrate recognition [17]. 

In MT13-C, the Post I domain includes all residues involved in the first two beta strands, as well as sections of their downstream loop structures [26]. The Post II domain has been identified in MT13-C as the region downstream of beta strand 4 and is thought to be involved in substrate recognition [17]. An excess of SAH has been shown to decrease the activity of METTL13, suggesting that SAH could function as a competitive inhibitor for METTL13, as it does for several other methyltransferases [17]. Specific amino acids identified to be potentially involved in substrate recognition include Asp575, Asp577, and Asn647 [26]. In addition, Gly503 and Glu524 have been identified as coordinating SAH, in a region analogous to S-adenosylmethionine coordination in spermidine synthase [8,17]. A schematic block diagram showing the sites is shown in Figure 1B. To date, the structural conformation of MT13-N, as well as full-length METTL13, has only been predicted using protein folding software such as AlphaFold, so the crystallization of MT13-N and full-length METTL13 in complex with SAM, SAH, or substrates is warranted to facilitate identification of characteristic structural features. 

The N-terminal methylation of eEFIA has been observed in yeasts and humans, suggesting that this modification is important in eEFIA regulation [11]. As stated above, all identified NTMTs in the human proteome have a 7βS structure: MT13-C, N-terminal methyltransferase 1 and 2 (NTMT 1 and 2 respectively), and METTL11 [11]. Most NTMTs have a recognition motif of X-P-[K/R], resulting in N-terminal lysine or arginine methylation. The recognition motif for MT13-C has been identified as M-[GAP]-[KRFYQH]-E-[KRQHIL], suggesting 49 possible targets for MT13-C in the human proteome [11,17]. Subsequent in vitro study of peptides derived from the proteins containing the identified binding motif were unable to validate any novel sites of methylation catalyzed by MT13-C, indicative of a possible high level of stringent specificity by MT13-C [17]. 

As stated previously, N-terminal methylation can occur on the first amino acid in any peptide sequence synthesized intracellularly, typically methionine. Alternatively, it can be removed by methionine aminopeptidases, allowing the amino group of the following residue to be targeted by NTMTs. The latter is the case for eEFIA methylation by MT13-C, as methylation occurs on the N-terminal of glycine 2 [11]. Orphan MT13-C can methylate the N-terminal of full length eEFIA, and it has been shown that this subunit can also catalyze the methylation of peptides, including the recognition motif, suggesting that the sequence is significant in substrate docking, as opposed to the structure [17]. The structure of MT13-C in complex with SAH has been crystalized as PDB 5WCJ (https://www.rcsb.org/structure/5wcj, accessed on 3 June 2024) [17], and subsequently docking experiments were carried out with the hexapeptide GKEKTH, both supporting the suggested mechanism of SAM/SAH and substrate binding [17].

Lysine methylation is a well-defined, reversible modification observed in both histones and non-histone targets. Instances of lysine methylation are now widespread, and it is known that this modification is involved in the regulation of numerous pathways in different disease states. The N-terminal domain of METTL13, MT13-N, catalyzes the trimethylation of eEFIA lysine 55, and it is generally less understood than MT13-C. Orphan MT13-N can methylate full-length eEFIA-K55 in vitro; however, to date, in vitro methylation of a peptide form of eEFIA-K55 has not been validated, suggesting that perhaps MT13-N requires more higher level structural information to catalyze methylation compared to MT13-C [17,25]. It has been shown that the occupancy levels of dimethylated lysine 55 of eEFIA, hereby referred to as eEFIA-K55me2, are higher in KRAS-driven cancers, and appear to be essential to meeting the increased translational output required for promoting tumorigenesis [25]. eEFIA1-K55 is located on the GTPase catalytic surface of eEFIA; therefore, the methylation of K55 is thought to modulate the GTPase activity directly. It has since been demonstrated that the presence of eEFIA-K55me2 increased the GTPase catalytic efficiency of eEFIA by roughly 20% [25]. It was also shown that when METTL13 is depleted in model cell lines, global protein synthesis is decreased, indicating the eEFIA-K55me2 is directly involved in protein synthesis modulation [25]. In general, although MT13-N bears more structural resemblance to known 7βS KMTs, its structure, function, and activity are less understood than those of MT13-C.

As previously stated, METTL13 was initially given the name FEAT, as it is faintly expressed in healthy tissues, and aberrantly expressed in tumors [19,26]. METTL13 expression has been identified as slightly elevated in normal testis [26]; however, in general, it has been shown that modulating METTL13 in healthy tissues has little to no effect on cell proliferation and survival [17,25], suggesting that METTL13 does not have a fundamental role in normal biology in healthy cells. Despite this, the research area surrounding METTL13 remains limited due to challenges associated with protein expression and the in vitro study of the enzyme. 

## 3. METTL13 Regulation

The expression of METTL13 is regulated at the transcriptional, translational, and post-translational levels. At the transcriptional level, cases of METTL13 regulation are present in a number of different disease states, to be discussed in a case-by-case basis later in this review. 

At the translational level, METTL13 expression is regulated by microRNAs (miRNAs). METTL13 was identified as a human homolog for a suppressor of rat neuronal apoptosis [27], and therefore, the role of METTL13 in the context of apoptosis was an area of initial study. A common proapoptotic miRNA, miR-16, was identified as an upstream regulator of METTL13 expression [27]. Liang et al. demonstrated that METTL13 was overexpressed at the protein level in various cancer tissues relative to their non-cancerous counterparts; however, at the mRNA level, the level of overexpression was much less substantial, suggesting that METTL13 expression may be regulated at a post-transcriptional level, a function consistent with that of many miRNAs [27]. Based on computational binding, modelled between common miRNAs and METTL13 mRNA, it was suggested that a binding interaction between miR-16 and the 3’-UTR of METTL13 mRNA was possible both through base-pairing and thermodynamically [27]. Liang et al. subsequently demonstrated that in the context of cancer cell lines, the overexpression of miR-16 resulted in a decrease in METTL13 expression at the protein level [27]. 

At the post-translational level, METTL13 forms a regulatory complex with two other known N-terminal methyltransferases, METTL11A and METTL11B, also known as NTMT1 and NTMT2, respectively [28]. For context, METTL11A can exist as a monomer or a dimer, and it catalyzes the trimethylation of a variety of protein N-terminals, while METTL11B exists as a monomer and catalyzes the monomethylation of different protein N-terminals, following methionine cleavage. The dimerization of METTL11A provides stability to the enzyme, and the formation of a heterotrimer between a METTL11A dimer and METTL11B provides further complex stability [28]. Aside from a strictly structural interaction, the formation of the METTL11A/METTL11B heterotrimer decreases the substrate specificity of METTL11A, allowing for more non-canonical substrates to be targeted by the enzyme. The physical interaction and resulting regulatory axes between different MTases are common within RNA and DNA methyltransferases [28], and Parker and Tooley sought to determine if METTL13 were involved in the regulatory pathway as well. Their work demonstrated that METTL11A, regardless of METTL11B involvement, had a non-catalytic interaction with METTL13, which resulted in mutual regulation [28]. 

The complexation of METTL13 with METTL11A inhibits the trimethylation activity catalyzed by the METTL11A and results in both an increase in Km and a decrease in V_max_ [28]. The same complex formation has opposing regulatory effects on METTL13 activity. METTL11A binding inhibits the trimethylation of the eEF1A N-terminal, with no significant effect on Km and a decrease in V_max_, suggesting that the interaction may be that of a standard non-competitive inhibitor [28]. In contrast, METTL11A complexation with METTL13 promotes the dimethylation of eEF1A K55, the canonical target of the MT13-N [28]. Although the results of the complex formation affect the catalytic efficiency of both enzymes, a catalytically active enzyme is not needed to elicit the regulatory effect on the other [28]. 

To further study how the two catalytic domains of METTL13 are differentially involved in the METTL11A/METTL13 complex, truncated versions of the enzyme were constructed, where residues 1 through 344 were used to represent the N-terminal catalytic domain, MT13-N, and residues 345 through 699 were used to represent the C-terminal catalytic domain, MT13-C [28]. Co-immunoprecipitation experiments conducted by Parker and Tooley demonstrated that MT13-N, which catalyzes the dimethylation of eEFIA-K55, interacts with METTL11A directly.

Finally, to determine if a METTL11A/METTL11B/METTL13 regulatory complex exists, the regulatory effect of all interactions was studied both in the presence and absence of METTL11A. This demonstrated that there is no direct interaction between METTL13 and METTL11B. Additionally, the inhibitory effect of METTL13 binding to METTL11A outcompetes the increased substrate promiscuity effects of the METTL11A/METTL11B heterotrimer [28]. The authors do note that the cellular localization of METTL13 and METTL11B are different. METTL13 is primarily cytoplasmic, whereas METTL11B is nuclear, suggesting that the opposing regulatory effects on METTL11A of the different methyltransferases have functionalities in different organelles; however, in the case where a METTL11A/METTL11B/METTL13 complex is formed, the effects of METTL13 on the METTL11A activity are dominant [28].

## 4. METTL13 in Disease

**Figure 2:**
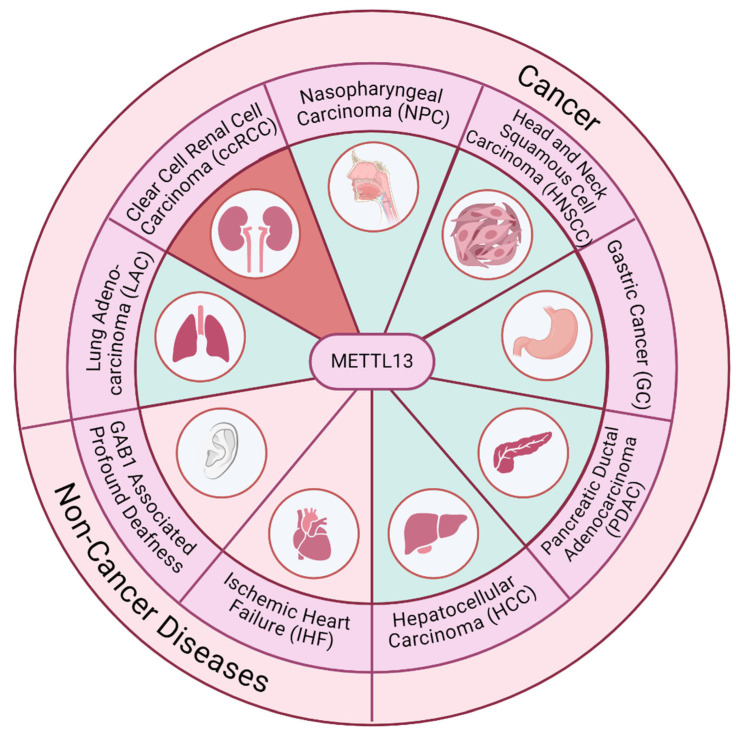
Visual summary of METTL13 involvement in various disease states. Instances of METTL13 overexpression are shown in green, and instances of METTL13 under-expression are shown in red. Created with Biorender.Com.

METTL13 and methylated eEFIA have been shown to be involved in several different cancers and other disease states, making METTL13 a promising therapeutic target. This section encompasses a contextual overview of the current scope of METTL13 involvement in disease pathways, and how disease phenotypes are affected by targeting METTL13. Most literature regarding METTL13 in disease focuses on identification of METTL13 as a biomarker for disease prognosis; however, more recent literature has proposed biochemical pathways for METTL13 in disease prognosis. A visual summary of METTL13 involvement in disease states is shown in Figure 2.

### 4.1. Pancreatic Ductal Adenocarcinoma (PDAC)

METTL13 is an oncogene in pancreatic ductal adenocarcinoma, hereby referred to as PDAC. An upregulation of METTL13 and eEFIA-K55me2 was observed in KRAS-driven PDAC [25]. In PDAC cells harboring catalytically inactive METTL13G58R, a decrease in eEFIA-K55me2 occupancy was observed, likely due to the mutation’s effect on SAM binding [25]. An in vivo study first showed that rearing METTL13-depleted mice did not affect the developmental or physiological mouse viability, showing again that METTL13 is faintly expressed in healthy tissue [25]. An in vitro study using the PDAC cell line demonstrated that depletion of METTL13 inhibited the formation of hallmark duct-like structures, a preliminary stage of PDAC prognosis referred to as acinar-to-ductal metaplasia [25]. Further in vivo studies, where KRAS-driven PDAC could be externally induced via caerulin injections to cause severe acute pancreatitis, showed that mice with METTL13-depleted mutations had more healthy pancreatic tissue and smaller tumor volumes compared to the control mice of the same age. Mice with the METTL13 deletion also had a greater median life expectancy when compared to the control group [25]. To date, the pathway by which METTL13-catalyzed methylation of eEFIA-K55me2 participates in tumorigenesis in PDAC is unknown, aside from generally increasing the global rate of protein synthesis [25]. However, it has been continuously demonstrated that METTL13 is involved in poor KRAS-driven PDAC prognosis, and that targeting METTL13 by removal leads to improvements in prognosis. 

### 4.2. Lung Adenocarcinoma (LAC)

METTL13 is an oncogene in lung adenocarcinoma, hereby referred to as LAC. Tumor growth typically involves KRAS activation [25], implicating METTL13 and eEFIA in disease prognosis. Normal expression and METTL13-depleted mutant mice were injected with an adenovirus, resulting in the expression of KRAS, and 16 weeks post-injection the control mice displayed widespread adenocarcinomas, as well as an upregulation of METTL13 and eEFIA-K55me2 [25]. Mutant mice, which were originally depleted of METTL13, did not show occupancy of eEFIA-K55me2 and showed significantly less tumor progression compared to the mice who originally had normal METTL13 expression [25]. In patient-derived xenografts (PDXs) from LAC, the depletion of METTL13 drastically reduced tumor growth, and supplementation of METTL13-depleted cells with the wild-type, catalytically active METTL13, restored occupancy of eEFIA-K55me2, resulting in increased tumor growth [25]. Supplementation of modified LAC PDX cells with catalytically inactive METTL13G58R had no effect on the rate of tumor growth and did not increase occupancy of eEF1A-K55me2, suggesting that the catalytically active METTL13 has an essential role in KRAS-driven LAC tumor growth [25]. 

### 4.3. Hepatocellular Carcinoma (HCC)

METTL13 is an oncogene in hepatocellular carcinoma, hereby referred to as HCC. Overexpression of METTL13 in HCC is associated with poor prognosis via the AP-2γ/METTL13/TCF3-ZEB1 transcriptional axis in various HCC cell lines [29]. Hematological and neurological expressed sequence 1-like gene (HNIL) is overexpressed in lung cancer, pancreatic cancer, and hepatocellular carcinoma (HCC). Li et al. demonstrated that the overexpression of HNIL led to the upregulation of the transcription factor AP-2γ (AP-2γ), leading to increased transcription of METTL13. It was suggested that AP-2γ binds the METTL13 promoter region, resulting in upregulated transcription. Following translation, METTL13 is then predicted to bind the transcription factor c-Myc directly, resulting in the upregulation of the transcription factor 3 (TCF3) and zinc-finger E-box binding homeobox 1 (ZEB1), all of which are known to be involved in tumor growth and metastasis [29]. The HNIL-induced upregulation of METTL13 was silenced by siRNA in HCC cell lines, resulting in a decrease in tumor progression and metastasis [29]. There is very limited data on METTL13 activity in HCC, so further research, focusing on METTL13 specifically is warranted to validate how METTL13 is involved in the proposed transcriptional axis, which currently excludes its modulation of eEFIA. 

### 4.4. Clear Cell Renal Cell Carcinoma (ccRCC)

Contrary to the examples above in which METTL13 acts as an oncogene, METTL13 is under-expressed in clear cell renal cell carcinomas (ccRCCs), and this under-expression is associated with disease progression [30]. It was suggested that in ccRCC, METTL13 downregulates the PI3K/AKT/mTOR/HIF-1α pathway, as well as the expression of c-Myc, both of which are involved in tumor growth [30]. A correlational analysis showed that there exists a negative relationship between METTL13 expression and tumor grade for ccRCC. This means that, as METTL13 expression increases, disease prognosis improves [30]. Immunohistochemical validation demonstrated that low levels of METTL13 expression are in fact associated with ccRCC, and that increasing METTL13 expression improved disease state by inhibiting tumor growth, metastasis, and endothelial-mesenchymal transition (EMT) [30]. Specifically, it was shown that silencing METTL13 in ccRCC cell lines affected levels of HIF-1α activity, and that the interaction was likely a METTL13-catalyzed PTM of HIF-1α, although this substrate relationship has not been substantiated [30]. METTL13 overexpression was also correlated with an increase in the phosphorylation of PI3K, AKT, and mTOR, all of which are involved in the HIF-1α translational pathway [30]. In vivo, when a ccRCC cell line with either standard or upregulated METTL13 were injected into mice, the tumors from the control cells were larger in size and weight, and the expression of two proteins involved in tumor growth, HIF-1α and c-Myc, was greater, suggesting that METTL13 acts as an inhibitor of tumor growth via both the PI3K/AKT/mTOR/HIF-1α- and c-Myc-driven pathways [30]. 

### 4.5. Gastric Cancer (GC)

METTL13 is an oncogene in gastric cancer, hereby referred to as GC [31]. Wu et al. showed that METTL13 was upregulated at both the mRNA and protein levels in various GC cell lines when compared to their non-cancerous counterparts. The same result was observed when using tissue samples from GC patients, compared to those from healthy volunteers [31]. The silencing of METTL13 resulted in a decrease in the tumorigenicity of GC cell lines, as shown by a decrease in cell proliferation, migration, and motility. The opposite effect on tumorigenicity was observed when METTL13 was overexpressed in GC cell lines [31], further supporting the role of METTL13 as an oncogene in GC. 

To uncover the molecular mechanism of METTL13 as an oncogene in GC, Wu et al. observed a positive correlation between both HN1L and METTL13 mRNA and protein expression levels. They demonstrated that HN1L mRNA was upregulated in GC cells overexpressing METTL13 but downregulated in GC cells with METTL13 silenced. In addition, they observed that silencing HN1L decreased GC cell proliferation [31], suggesting that HN1L may play an oncogenic role as well. The canonical target of METTL13, eEF1A, was then silenced and, as a result, it was observed that HN1L expression decreased at both the mRNA and protein levels, suggesting a possible METTL13/eEF1A/HN1L regulatory pathway in GC [31]. Site mutation of the known METTL13-catalyzed lysine in eEF1A, K55 to A55, did not affect the expression level of HN1L, however, leading the authors to suggest that the regulation of HN1L by METTL13 and eEF1A exists in a methylation-independent manner [31]. It was demonstrated as well that HN1L expression promotes METTL13 expression, suggesting that a METTL13/eEF1A/HN1L feedback loop may also exist [31], although additional research to characterize the exact molecular mechanisms of action is needed. 

### 4.6. Nasopharyngeal Cancer (NPC)

METTL13 has an oncogenic role in nasopharyngeal carcinoma, hereby referred to as NPC. As with many other cancers, the upregulation of METTL13 is correlated with poor patient prognosis in NPC. It was demonstrated that in NPC cell lines, the expression levels of METTL13 are elevated when compared to their non-cancerous counterparts, and that the silencing of METTL13 in NPC cell lines results in decreased cell proliferation, migration speed, and invasiveness, as well as in an antiangiogenetic effect [32]. 

It was shown that the involvement of METTL13 in NPC typically follows the METTL13/ZEB1/TCT1 axis. It was hypothesized by the authors that, based on database information, there may be an interaction between METTL13 and TPT1, which is a transcription factor upregulated in NPC relative to non-cancerous counterparts. Ni et al. demonstrated that elevated METTL13 expression in NPC results in the upregulation of ZEB1, a known transcription factor involved in EMT in a variety of different cancers. ZEB1 was then suggested to have a high affinity for the TCT1 promoter region, resulting in an increase in TPT1 transcription and expression [32]. It was hypothesized that an increased expression of TPT1 results in an increase in tumorigenesis. This suggests that METTL13 is involved in the disease pathway as an upstream regulator, as opposed to a main oncogene. In NPC cell lines with silenced METTL13 genes, an overexpression of TPT1 restored the disease state, suggesting that overexpression of TPT1 may reverse the antitumor effects of METTL13 silencing [32]. 

It is of note as well that METTL13 overexpression in NPC affected the expression levels of proteins outside the METTL13/ZEB1/TPT1 axis. In METTL13 KO NPC cell lines, a decrease in MMP2 and MMP9 protein expression levels was observed [32]. Overall, Ni et al. demonstrated the involvement of METTL13 in NPC progression via the METTL13/ZEB1/TPT1 axis and suggested additional proteins which may be regulated by METTL13 in NPC. 

### 4.7. Head and Neck Squamous Cell Carcinoma (HNSCC)

METTL13 is an oncogene in head and neck squamous cell carcinoma, hereby referred to as HNSCC [33]. An overexpression of METTL13 at both the mRNA and protein levels is observed in HNSCC cell lines relative to their non-cancerous counterparts, and silencing METTL13 results in decreased cell proliferation, wound healing abilities, migration and sphere-forming capabilities, as well as an increase in apoptosis, suggesting elevated METTL13 levels are correlated with poor prognosis [33]. 

Wang et al. connected METTL13 with the transcription factor Snail, which is known to be involved in EMT signaling pathways in HNSCC. HNSCC cells with increased expression of Snail are more motile and less adherent, making them more prone to tumor metastasis. The depletion of METTL13 in HNSCC cell lines resulted in an overall decrease in global protein synthesis [33], which may be due to the canonical targeting of eEFIAK55, which has been demonstrated to increase the rate of mRNA translation in other cancer types. The depletion of METTL13 in HNSCC cell lines also resulted in the general downregulation of EMT signaling pathways, and there was a downregulation of Snail at the mRNA level, suggesting that METTL13 may be involved in Snail regulation at a transcriptional level [33]. It was demonstrated as well that restoring Snail expression levels in HNSCC METTL13 KO cell lines reversed the antitumor effects of METTL13 silencing [33]. Although a clear signaling pathway has not yet been determined involving METTL13 in HNSCC, it was clearly shown that a METTL13/Snail axis exists and is involved in disease prognosis, warranting additional research.

Although it is important to note that instances of METTL13 upregulation resulting in improvements in disease phenotypes exist [30], they are less prevalent and less validated, and primary literature about this property of METTL13 has been retracted, specifically findings suggesting that upregulation of METTL13 resulted in improvements in disease phenotypes for bladder carcinomas. As the proposed research aims to identify drug candidates for METTL13, disease states in which the upregulation of METTL13 results in poor prognosis are of greater interest.

### 4.8. METTL13 in Other Disease Indications

Beyond cancer, the function of METTL13 has been explored and implicated in a number of disease indications. In ischemic heart failure (IHF), Yu et al. proposed a novel site of lysine methylation catalyzed by METTL13 and a resulting PTM-regulated pathway involved in the pathology of heart failure as a result of myocardial infarction [34]. As other members of the METTL protein family, including METTL3 and METTL14, have been identified in heart diseases, researchers sought to investigate a potential role of METTL13, due to the known role it plays in regulating translation rates in cancers [19]. A transport protein involved in maintaining calcium ion homeostasis in cardiomyocytes, sarcoplasmic reticulum calcium ATPase 2a (SARCA2a), has been shown to be downregulated at a protein expression level post myocardial infarction, and it is thought to contribute to negative prognosis, leading to heart failure. Replenishing SARCA2a expression and activity levels has also been shown to restore the negative effects on calcium ion homeostasis and contractile function caused by its downregulation [34]. SARCA2a is known to be modulated by several PTMs, making the identification of its upstream regulators a potential avenue for developing IHF therapeutics. The downregulation of METTL13 has been identified in tissue samples from cardiomyopathy, and therefore Yu et al. sought to determine whether METTL13 plays a role in IHF pathology. 

It was shown that METTL13 was downregulated at both the molecular and protein levels following myocardial infarction (MI), and that the overexpression of METTL13 improved the calcium ion homeostasis and contractile function of post-MI cardiomyocytes, as well as downregulated the expression of known heart failure marker genes [34]. Yu et al. demonstrated as well that silencing METTL13 in healthy cardiomyocytes introduced dysfunction in calcium ion homeostasis and a decrease in contractility, two parameters identified in MI tissues. Overexpression of METTL13 was shown to increase SERCA2a expression at a protein level, but not at the mRNA level, suggesting that an interaction pathway may exist and involves additional proteins [34]. The downregulation of SERCA2a because of METTL13 silencing was then shown to be restored upon the introduction of a general ubiquitin-proteasome inhibitor, demonstrating that silencing METTL13 increases the ubiquitination of SERCA2a, a preliminary step in signaling protein degradation [34]. Several known E3 ubiquitin–protein ligases were investigated, and it was shown that the expression level of Casitas B-lineage lymphoma, hereby referred to as c-Cbl, was dependent on that of METTL13. Specifically, the overexpression of METTL13 resulted in the downregulation of c-Cbl expression at the protein level [34]. Knock-down experiments conducted by Yu et al. demonstrated that knocking down c-Cbl resulted in an increase in SERCA2a expression and a decrease in SERCA2a ubiquitination, indicating that c-Cbl may be responsible for the ubiquitination of SERCA2a [34]. It was shown as well that METTL13 overexpression resulted in a downregulation of c-Cbl, and therefore, a decrease in SERCA2a ubiquitination, resulting in less SERCA2a protein degradation and an improvement in the outlined disease characteristics of MI. c-Cbl is regulated by PTM, and it was identified that lysine methylation of c-Cbl was dependent on the presence of enzymatically active METTL13, giving rise to a proposed METTL13/c-Cbl/SERCA2a axis [34]. 

METTL13 has also been implicated in GAB1-associated profound deafness. The METTL13 locus is on chromosome one, specifically 1q24, an area in the same meiotic locus as the deafness-associated gene, DFNM1, which encodes a dominant suppressor of DFNB26 recessive, which is profound deafness [35]. A missense mutation in METTL13, c.1631G > A, which encodes p.Arg544Gln, results in an arginine at site 544 in the MT13-C catalytic domain. Yousef et al. identified that within their sample, individuals experiencing profound deafness were homozygous for wild-type METTL13, suggesting that p.Arg544Gln may be a dominant site mutation [35]. It was then demonstrated, using zebrafish as a model organism, that the p.Arg544Gln mutation suppressed DFNB26 deafness associated with a mutated GAB1 variant [35]. GAB1 is a protein involved in several signaling pathways in the inner ear and is generally thought to be associated with auditory signaling pathways [35]. Additionally, Yousef et al. identified a GAB-1 modifier variant, in which a site mutation for GAB-1 resulted in misfolding in the protein scaffold, resulting in profound deafness. Co-immunoprecipitation experiments conducted by Yousef et al. showed that METTL13, GAB-1, and an additional protein involved in auditory signaling, SPRY2, precipitated together, suggesting that METTL13, along with GAB-1, may be involved in inner ear signaling [35]. This work focuses on modifier variants and their role in disease onset; therefore, to further elucidate the role of METTL13 in auditory processes, it is essential to examine the effects of the site mutation on METTL13 N-terminal methylation activity and the resulting molecular pathways.

## 5. Conclusions

Since the identification of METTL13 as an upregulated immunogenic protein in a variety of different cancer pathways [19], the literature has continued to implicate METTL13 in broad disease progression and begun uncovering its molecular mechanisms of action. Areas of future study for METTL13 include uncovering novel target sites of lysing and N-terminal methylation, exploring their potential roles in general cell pathways, as well as disease progression, and conducting structural research to determine the exact molecular mechanisms of methylation for both catalytic domains. In addition, METTL13 should be investigated as a therapeutic target for drug development.

## References

[1] Ramazi S., Zahiri J. (2021). Post-Translational Modifications in Proteins: Resources, Tools and Prediction Methods. Database.

[2] Deribe Y.L., Pawson T., Dikic I. (2010). Post-Translational Modifications in Signal Integration. Nat. Struct. Mol. Biol..

[3] Li W., Li F., Zhang X., Lin H., Xu C. (2021). Insights into the Post-Translational Modification and Its Emerging Role in Shaping the Tumor Microenvironment. Signal Transduct. Target. Ther..

[4] Liu R., Zhao E., Yu H., Yuan C., Abbas M.N., Cui H. (2023). Methylation across the Central Dogma in Health and Diseases: New Therapeutic Strategies. Signal Transduct. Target. Ther..

[5] Bhat K.P., Ümit Kaniskan H., Jin J., Gozani O. (2021). Epigenetics and beyond: Targeting Writers of Protein Lysine Methylation to Treat Disease. Nat. Rev. Drug Discov..

[6] Poulard C., Corbo L., Le Romancer M. (2016). Protein Arginine Methylation/Demethylation and Cancer. Oncotarget.

[7] Wu Q., Schapira M., Arrowsmith C.H., Barsyte-Lovejoy D. (2021). Protein Arginine Methylation: From Enigmatic Functions to Therapeutic Targeting. Nat. Rev. Drug Discov..

[8] Jakobsson M.E. (2021). Structure, Activity and Function of the Dual Protein Lysine and Protein N-terminal Methyltransferase Mettl13. Life.

[9] Kapell S., Jakobsson M.E. (2021). Large-Scale Identification of Protein Histidine Methylation in Human Cells. NAR Genom. Bioinform..

[10] Sprung R., Chen Y., Zhang K., Cheng D., Zhang T., Peng J., Zhao Y. (2008). Identification and Validation of Eukaryotic Aspartate and Glutamate Methylation in Proteins. J. Proteome Res..

[11] Huang R. (2019). Chemical Biology of Protein N-Terminal Methyltransferases. ChemBioChem.

[12] Chen P., Huang R., Hazbun T.R. (2023). Unlocking the Mysteries of Alpha-N-Terminal Methylation and Its Diverse Regulatory Functions. J. Biol. Chem..

[13] Falnes P., Małecki J.M., Herrera M.C., Bengtsen M., Davydova E. (2023). Human Seven-β-Strand (METTL) Methyltransferases—Conquering the Universe of Protein Lysine Methylation. J. Biol. Chem..

[14] Schnee P., Pleiss J., Jeltsch A. (2024). Approaching the Catalytic Mechanism of Protein Lysine Methyltransferases by Biochemical and Simulation Techniques. Crit. Rev. Biochem. Mol. Biol..

[15] Trievel R.C., Beach B.M., Dirk L.M.A., Houtz R.L., Hurley J.H. (2002). Structure and Catalytic Mechanism of a SET Domain Protein Methyltransferase. Cell.

[16] Min J., Feng Q., Li Z., Zhang Y., Xu R.-M., Keck W.M. (2003). Structure of the Catalytic Domain of Human DOT1L, a Non-SET Domain Nucleosomal Histone Methyltransferase. Cell.

[17] Jakobsson M.E., Małecki J.M., Halabelian L., Nilges B.S., Pinto R., Kudithipudi S., Munk S., Davydova E., Zuhairi F.R., Arrowsmith C.H. (2018). The Dual Methyltransferase METTL13 Targets N Terminus and Lys55 of EEF1A and Modulates Codon-Specific Translation Rates. Nat. Commun..

[18] Abbas W., Kumar A., Herbein G. (2015). The EEF1A Proteins: At the Crossroads of Oncogenesis, Apoptosis, and Viral Infections. Front. Oncol..

[19] Takahashi A., Tokita H., Takahashi K., Takeoka T., Murayama K., Tomotsune D., Ohira M., Iwamatsu A., Ohara K., Yazaki K. (2011). A Novel Potent Tumour Promoter Aberrantly Overexpressed in Most Human Cancers. Sci. Rep..

[20] Li Y., Kobayashi K., Mona M.M., Satomi C., Okano S., Inoue H., Tani K., Takahashi A. (2016). Immunogenic FEAT Protein Circulates in the Bloodstream of Cancer Patients. J. Transl. Med..

[21] Conery A.R., Rocnik J.L., Trojer P. (2022). Small Molecule Targeting of Chromatin Writers in Cancer. Nat. Chem. Biol..

[22] Honma D., Kanno O., Watanabe J., Kinoshita J., Hirasawa M., Nosaka E., Shiroishi M., Takizawa T., Yasumatsu I., Horiuchi T. (2017). Novel Orally Bioavailable EZH1/2 Dual Inhibitors with Greater Antitumor Efficacy than an EZH2 Selective Inhibitor. Cancer Sci..

[23] Stein E.M., Garcia-Manero G., Rizzieri D.A., Tibes R., Berdeja J.G., Savona M.R., Jongen-Lavrenic M., Altman J.K., Thomson B., Blakemore S.J. (2018). The DOT1L Inhibitor Pinometostat Reduces H3K79 Methylation and Has Modest Clinical Activity in Adult Acute Leukemia. Blood.

[24] Izutsu K., Makita S., Nosaka K., Yoshimitsu M., Utsunomiya A., Kusumoto S., Morishima S., Tsukasaki K., Kawamata T., Ono T. (2023). An Open-Label, Single-Arm Phase 2 Trial of Valemetostat for Relapsed or Refractory Adult T-Cell Leukemia/Lymphoma. Blood.

[25] Liu S., Hausmann S., Carlson S.M., Fuentes M.E., Francis J.W., Pillai R., Lofgren S.M., Hulea L., Tandoc K., Lu J. (2019). METTL13 Methylation of EEF1A Increases Translational Output to Promote Tumorigenesis. Cell.

[26] Nagase T., Ishikawa K.-I., Suyama M., Klkuno R., Hlrosawa M., Miyajima N., Tanaka A., Kotani H., Nomura N., Ohara O. (1998). Prediction of the Coding Sequences of Unidentified Human Genes. XII. The Complete Sequences of 100 New CDNA Clones from Brain Which Code for Large Proteins In Vitro. DNA Res..

[27] Liang H., Fu Z., Jiang X., Wang N., Wang F., Wang X., Zhang S., Wang Y., Yan X., Guan W. (2015). MiR-16 Promotes the Apoptosis of Human Cancer Cells by Targeting FEAT. BMC Cancer.

[28] Parker H.V., Schaner Tooley C.E. (2023). Opposing Regulation of the Nα-Trimethylase METTL11A by Its Family Members METTL11B and METTL13. J. Biol. Chem..

[29] Li L., Zheng Y.L., Jiang C., Fang S., Zeng T.T., Zhu Y.H., Li Y., Xie D., Guan X.Y. (2019). HN1L-Mediated Transcriptional Axis AP-2γ/METTL13/TCF3-ZEB1 Drives Tumor Growth and Metastasis in Hepatocellular Carcinoma. Cell Death Differ..

[30] Liu Z., Sun T., Piao C., Zhang Z., Kong C. (2021). METTL13 Inhibits Progression of Clear Cell Renal Cell Carcinoma with Repression on PI3K/AKT/MTOR/HIF-1α Pathway and c-Myc Expression. J. Transl. Med..

[31] Wu Q., Hu Q., Hai Y., Li Y., Gao Y. (2023). METTL13 Facilitates Cell Growth and Metastasis in Gastric Cancer via an EEF1A/HN1L Positive Feedback Circuit. J. Cell Commun. Signal.

[32] Ni H., Liang C., Zhou Z., Jiang B., Li Y., Shang H., Yu X. (2023). METTL13 Promotes Nasopharyngeal Carcinoma Progression through Regulating the ZEB1/TPT1 Axis. J. Gene Med..

[33] Wang X., Li K., Wan Y., Chen F., Cheng M., Xiong G., Wang G., Chen S., Chen Z., Chen J. (2021). Methyltransferase like 13 Mediates the Translation of Snail in Head and Neck Squamous Cell Carcinoma. Int. J. Oral. Sci..

[34] Yu S., Sun Z.Y., Wang X., Ju T., Wang C., Liu Y., Qu Z., Liu K.W., Mei Z., Li N. (2023). Mettl13 Protects against Cardiac Contractile Dysfunction by Negatively Regulating C-Cbl-Mediated Ubiquitination of SERCA2a in Ischemic Heart Failure. Sci. China Life Sci..

[35] Yousaf R., Ahmed Z.M., Giese A.P.J., Morell R.J., Lagziel A., Dabdoub A., Wilcox E.R., Riazuddin S., Riazuddin S., Friedman T.B. (2018). Modifier Variant of METTL13 Suppresses Human GAB1-Associated Profound Deafness. J. Clin. Investig..

